# Luteinizing hormone activates the Hippo pathway to promote progesterone synthesis in bovine luteal cells

**DOI:** 10.1186/s12964-026-02917-w

**Published:** 2026-05-02

**Authors:** Farzaneh Tamanaeifar, Bunmi A. Owolabi, Corrine F. Monaco, Robyn M. Moses, Ailenn C. Castillo, Andrea S. Cupp, John S. Davis, Michele R. Plewes

**Affiliations:** 1 Department of Genetics, Cell Biology and Anatomy, University of Nebraska Medical Center, 985870 Nebraska Medical Center, Omaha, NE 68198-5870 USA; 2 Department of Obstetrics and Gynecology, Olson Center for Women’s Health, University of Nebraska Medical Center, 983255 Nebraska Medical Center, Omaha, NE 68198-3255 USA; 3 Department of Cellular and Integrative Physiology, University of Nebraska Medical Center, 985870 Nebraska Medical Center, Omaha, NE 68198-5870 USA; 4 Department of Animal Sciences, University of Nebraska-Lincoln, Lincoln, NE 68583-0908 USA; 5 Veterans Affairs Nebraska Western Iowa Health Care System, 4101 Woolworth Ave, Omaha, NE 68105 USA; 6 Department of Biochemistry and Molecular Biology, University of Nebraska Medical Center, 985870 Nebraska Medical Center, Omaha, NE 68198-5870 USA

**Keywords:** Hippo signaling pathway, YAP1, TAZ, Corpus luteum, Progesterone, Luteinizing hormone (LH), Steroidogenesis, CAMP/PKA signaling, Reproductive endocrinology

## Abstract

**Background:**

Progesterone production by the corpus luteum is essential for embryo implantation and early pregnancy maintenance and is acutely stimulated by luteinizing hormone (LH). While LH signaling through protein kinase A (PKA) is well established, downstream regulatory networks that constrain or shape luteal steroidogenesis remain incompletely defined. Here, we identify a previously unrecognized role for the Hippo signaling pathway in regulating luteal progesterone production.

**Methods:**

Using primary bovine luteal cells isolated from corpora lutea, we examined the relationship between LH/PKA signaling and Hippo signaling pathway activity. Expression, phosphorylation, and subcellular localization of Hippo pathway components were assessed by immunoblotting and nuclear fractionation. Progesterone production was quantified by ELISA. LH-induced transcriptional responses were analyzed using upstream regulator prediction from RNA sequencing. Functional roles of YAP1 and TAZ were evaluated using adenoviral overexpression of constitutively active mutants and siRNA-mediated knockdown.

**Results:**

Hippo pathway components were enriched in luteal cells relative to follicular precursors. LH rapidly increased phosphorylation and cytoplasmic sequestration of YAP1 and TAZ in small luteal cells through PKA. Pharmacologic inhibition of LATS1/2 did not alter LH-stimulated progesterone production, suggesting that LH-induced steroidogenesis is not limited by LATS-dependent regulation of YAP1/TAZ. Sustained activation of YAP1 or TAZ suppressed LH-induced progesterone synthesis, whereas depletion of either factor enhanced progesterone output. Consistently, RNA-seq analysis identified YAP1/TAZ, and TEAD transcription factors as inhibited upstream regulators following LH stimulation in small luteal cells.

**Conclusions:**

Our findings support a model in which LH, via PKA and activation of Hippo signaling promotes progesterone synthesis by restraining YAP1/TAZ transcriptional activity in small luteal cells. This work identifies Hippo signaling as an unrecognized regulatory layer in luteal steroidogenesis and highlights YAP1/TAZ as potential therapeutic target for luteal insufficiency and infertility.

**Supplementary Information:**

The online version contains supplementary material available at 10.1186/s12964-026-02917-w.

## Introduction

The corpus luteum is a transient endocrine gland that forms from the remnants of the ovulated follicle and undergoes a rapid structural and functional transformation in response to luteinizing hormone (LH) [1]. This transformation involves the differentiation of granulosa and theca cells into steroidogenic large and small luteal cells, respectively, which coordinate a significant increase in progesterone synthesis, an essential hormone for establishing and maintaining early pregnancy [2]. While large luteal cells produce the majority of basal progesterone, small luteal cells are highly responsive to LH due to greater expression of LH receptors, highlighting LH’s central role in regulating dynamic steroidogenic output in the corpus luteum [3]. Accordingly, the present study focuses on small luteal cells as the primary LH-responsive model, while large luteal cells are included in defined experimental contexts to assess steroidogenesis under basal, LH-independent conditions. LH acts primarily through cAMP/Protein Kinase A (PKA) signaling cascades, which enhance cholesterol mobilization and its conversion to progesterone [4, 5]. Despite this well-characterized signaling axis, the mechanisms that restrain or fine-tune LH-mediated progesterone synthesis within small luteal cells remain poorly defined. Identifying these regulatory nodes is essential for understanding how the corpus luteum adapts to hormonal cues during the luteal phase and how dysregulation of these processes may contribute to luteal insufficiency and reproductive failure.

One candidate regulatory pathway is the Hippo signaling, a conserved regulatory network best known for constraining cell proliferation, apoptosis, and tissue homeostasis [6]. Core components of the Hippo pathway include MST1/2 (Mammalian STE20-like Protein Kinases 1 and 2), and LATS1/2 (Large Tumor Suppressor Kinases 1 and 2) kinases, which phosphorylate and inactivate the transcriptional co-activators YAP1 (Yes-Associated Protein 1) and TAZ (WW domain containing transcription regulator 1; also known as WWTR1). When the Hippo pathway is activated, YAP1 and TAZ are phosphorylated and sequestered in the cytoplasm, preventing them from entering the nucleus and initiating gene expression [7]. When Hippo signaling is inactive, YAP1 and TAZ translocate to the nucleus, where they partner primarily with TEAD transcription factors to regulate gene expression programs linked to cellular growth and metabolism [8].

Emerging evidence implicates the Hippo signaling pathway in regulating the metabolic and transcriptional networks that govern steroid hormone biosynthesis across multiple endocrine tissues [9, 10]. In hepatocytes, YAP1 enhances lipid and cholesterol biosynthesis by interacting with SREBP1/2 (Sterol regulatory element-binding proteins 1 and 2), promoting the transcription of key enzymes such as FASN (Fatty Acid Synthase) and HMGCR (3-Hydroxy-3-methylglutaryl-CoA reductase) [11]. Furthermore, previous work demonstrated that Hippo-YAP1 signaling regulates aromatase expression and estradiol production in KGN (human granulosa-like tumor) and primary human granulosa cells [12]. In a more recent study, it was shown that YAP1/TAZ-TEAD signaling suppresses the steroidogenic program, such that inhibition of YAP1/TAZ enhances expression of CYP19A1 (Cytochrome P450 Family 19 Subfamily A Member 1), CYP11A1 (Cytochrome P450 Family 11 Subfamily A Member 1), and HSD3B (3β-Hydroxysteroid Dehydrogenase) in KGN cells, while constitutively active YAP1 blunts the FSH-induced expression of steroidogenic genes in rat granulosa cells [13]. In Leydig cells, TAZ suppresses steroidogenesis by downregulating transcriptional enzymes critical for androgen production, such as STAR (Steroidogenic Acute Regulatory protein), CYP11A1, and HSD3B [14]. Collectively, these findings indicate that YAP1 and TAZ function as context-dependent regulators of steroidogenic capacity, with inhibitory roles described in multiple proliferative or hormone-responsive endocrine cell types. Whether this regulatory paradigm extends to terminally differentiated, non-proliferative luteal cells, which are specialized for sustained progesterone production, remains unclear. Specifically, it is unknown whether luteinizing hormone (LH) engages Hippo pathway components to regulate progesterone biosynthesis in the corpus luteum. Clarifying this intersection addresses a fundamental gap in our understanding of how growth-restraining pathways interface with hormone-driven steroidogenesis in differentiated endocrine tissues.

In this study, we investigated how LH signaling employs the Hippo pathway to promote steroidogenesis in small luteal cells. We demonstrate that LH stimulation activates Hippo signaling via the cAMP/PKA axis, resulting in increased phosphorylation and cytoplasmic retention of YAP1/TAZ, and enhanced progesterone production in small luteal cells. In contrast, genetic inactivation of Hippo signaling through overexpression of constitutively active YAP1/TAZ reduces LH-induced progesterone synthesis, suggesting that repression of YAP1/TAZ transcriptional activity is required for optimal steroidogenesis in small luteal cells. Our findings establish a previously unrecognized role for Hippo signaling as a permissive regulator of LH-driven progesterone production in luteal cells and identify YAP1/TAZ as critical nodes linking hormonal signaling to metabolic output in the corpus luteum.

### Reagents

Opti-MEM, M199 culture media, fetal bovine serum (FBS), Dulbecco’s modified Eagle’s medium (DMEM; calcium-free, 4.0 g/L glucose), phosphate-buffered saline (PBS), penicillin–streptomycin, and gentamicin sulfate were purchased from Gibco (Thermo Fisher Scientific, Waltham, MA, USA). Lipofectamine RNAiMAX Transfection Reagent and UltraPure™ TEMED were obtained from Invitrogen (Thermo Fisher Scientific, Carlsbad, CA, USA). Ultra-pure acrylamide (ProtoGel) was purchased from National Diagnostics (Atlanta, GA, USA). Nitrocellulose membrane, Bicinchoninic Acid (BCA) protein assay, RIPA buffer, Halt Protease and Phosphatase Inhibitor Cocktail, and SuperSignal West Femto chemiluminescent substrate were obtained from Pierce (Thermo Fisher Scientific, Rockford, IL, USA). Bovine luteinizing hormone (LH) was obtained from Tucker Endocrine Research Institute (Atlanta, GA, USA). U0126 (ERK inhibitor) was purchased from Tocris Bioscience (Minneapolis, MN, USA). Adenoviral vectors expressing constitutively active YAP1(S127A) or TAZ(S89A) were custom produced by Vector Biolabs using plasmids obtained from Addgene (#27370 and #24815). The resulting adenoviral human type 5 (dE1/E3) vectors were replication-deficient, with titers of 3.6 × 10^1^⁰ PFU/mL (Lot #20231226 T#7) and 5.4 × 10^1^⁰ PFU/mL (Lot #20231226 T#8), respectively. Control siRNA (siCTL; Red Glow-labeled siRNA, D-001630–02–05), targeting YAP1 (siYAP1, L-012200–00–0010), TAZ (siTAZ, L-016083–00–0010), and LATS1 (siLATS1, L-004632–00–0005) were purchased from Dharmacon (Horizon Discovery, Cambridge, UK). Progesterone ELISA kits were obtained from DRG International, Inc. (Springfield, NJ, USA). Nonfat milk and APS were sourced from Fisher scientific (Mt Prospect, IL, USA). Phos-tag Acrylamide was from FUJIFILM Wako Pure Chemical Corporation (Richmond, VA, USA). Penicillin G-sodium, streptomycin sulfate, HEPES, bovine serum albumin (BSA), deoxyribonuclease I, Tris–HCl, EDTA, EGTA, sodium fluoride, Na₄O₂O₇, Na₃VO₄, β-mercaptoethanol, sodium chloride, Triton X-100, glycerol, SDS, bromophenol blue, Tween-20, paraformaldehyde, magnesium chloride (MgCl₂), potassium chloride (KCl), Manganese chloride (MnCl_2_) and KT5720 were purchased from Sigma-Aldrich (St. Louis, MO, USA). Collagenase I and II were obtained from Worthington Biochemical Corporation (Lakewood, NJ, USA). TRULI was purchased from MedChemExpress (South Brunswick, NJ, USA). TRIzol® Reagent and the Direct-zol™ RNA Miniprep Kit were obtained from Zymo Research (Irvine, CA, USA).

## Methods

### Bovine follicular cell isolation

All procedures were approved by the University of Nebraska-Lincoln Institutional Animal Care and Use Committee and performed at the University of Nebraska-Lincoln Animal Science Department. Granulosa and theca cells were isolated from a cohort of post-pubertal, non-lactating multiparous beef cows (n = 6) of composite breeding (25% MARC III [1/4 Angus, 1/4 Hereford, 1/4 Pinzgauer, 1/4 Red Poll] and 75% Red Angus) from the beef physiology herd at the Eastern Nebraska Research, Education and Extension Center (ENREEC) as previously described [15]. Briefly, estrous cycles were synchronized using two injections of PGF2α (25 mg/mL; i.m.; Lutalyse, Zoetis Animal Health) 14 days apart, with estrus confirmed by heat patch activation (≥ 80%). A third injection of PGF2α was administered on days 9–11 at mid-cycle, and ovariectomy was performed via high lumbar incision [16] at 12 and 24 h after the third injection.

Granulosa and theca cells were isolated from the largest and second-largest antral follicles. Specifically, granulosa cells were collected by aspiration and gentle scraping of the follicular wall and suspended in DMEM/F12 medium. Cells were washed by centrifugation (150 × *g*, 5–10 min) and filtered through a 70 μm nylon mesh to remove debris. Following granulosa cell removal, the theca interna layer was mechanically isolated using fine forceps, enzymatically dispersed in collagenase II (103 IU/mL) in DMEM/F12, and incubated at 37 °C with agitation for 1 h. Dispersed theca cells were filtered through a 70 μm mesh and washed three times by centrifugation (150 × *g*, 5–10 min) [15].

### Follicular cells differentiation

Bovine granulosa and theca cells (both 1 × 10^6^ cells/well) were seeded in a 6-well dish and allowed to adhere overnight in DMEM/F12 medium containing 1% FBS and antibiotics (100 U/mL of penicillin and 100 μg/mL of streptomycin). Differentiation was induced the next day following an established protocol. Briefly, cultures were switched to differentiation medium [DMEM/F12, 1% FBS, insulin (10 mg/L), transferrin (5.5 mg/L), sodium selenium (6.7 μg/L), forskolin (FSK; 10 μM; adenylyl cyclase activator), phorbol myristate acetate (PMA, 20 nM; PKC/MAPK activator), and antibiotics] and maintained for 4 days. On day 2, cells were washed twice with warm PBS and replenished with fresh differentiation medium for an additional 48 h. Control wells received DMEM/F12 supplemented with 1% FBS with antibiotics only [15].

### Bovine luteal cell isolation

Corpus luteum tissue was collected from a separate cohort of post-pubertal, non-lactating multiparous beef cows (*n* = 6; same breed and herd as above). Estrous cycles were synchronized using two intramuscular injections of PGF2α (25 mg; Lutalyse, Zoetis Inc.) 11 days apart. At mid-cycle, on days 9–10 of the estrous cycle, cows were ovariectomized via high lumbar incision [16]. The corpus luteum was surgically dissected from the ovary, weighed, and transported to the laboratory on ice in basal medium M199.

Using sterile technique, the corpus luteum was finely minced with microtome and surgical scissors, and enzymatically dissociated using collagenase I (103 U/mL) in M199 [supplemented with antibiotics (100 U/mL penicillin G-sodium, 100 μg/mL streptomycin sulfate, and 10 μg/mL gentamicin sulfate)] for 40 min in spinner flasks at 37 °C. Following incubation, the supernatant was transferred to a sterile 50 mL culture tube, washed three times with elutriation medium (calcium-free DMEM, 4.0 g/L glucose, antibiotics, 25 mM HEPES, 0.1% bovine serum albumin (BSA), and 0.02 mg/mL deoxyribonuclease I; pH 7.4), resuspended in 15 mL of elutriation medium and placed on ice. Fresh dissociation medium was added to the remaining undigested tissue and incubated with agitation for an additional 40 min. The remaining cells were collected, washed twice with elutriation medium at 150 × *g* for 5 min each, and combined with the previous sample [17]. Following elutriation, cells were pelleted and resuspended in elutriation medium. Viability and concentration were determined using Trypan Blue exclusion and a hemocytometer. Cells with a diameter of 15–25 μm were classified as small luteal cells (purity > 90%), while those with a diameter > 30 μm were classified as large luteal cells (purity 25–50%).

### Cell preparation and treatments

Enriched populations of small and large luteal cell cultures were plated in 12-well culture dishes at 5 × 10^5^ and 2 × 10^5^ cells/well, respectively. Cells were cultured overnight in culture media (M199 supplemented with 5% FBS, 0.1% BSA, and Penicillin Streptomycin Solution) at 37 °C in an atmosphere of 95% humidified air and 5% CO2. Prior to the experiment, luteal cells were rinsed with PBS and equilibrated in fresh serum-free culture media (M199 supplemented with 0.1% BSA, and Penicillin Streptomycin Solution) for 2 h. To assess the effects of LH on the phosphorylation of Hippo pathway components, cells were treated with culture medium alone or LH (10 ng/mL) for up to 4 h. Following incubation, conditioned media was collected for progesterone measurements and cell lysates were collected for Western blot analysis.

For inhibitor studies, enriched small luteal cells were pretreated for 1 h with vehicle control (DMSO), KT5720 (PKA inhibitor; 10 µM), U0126 (MEK inhibitor; 10 µM), or TRULI (LATS1/2 inhibitor; 10 µM). After pretreatment, cells were stimulated with LH (10 ng/mL) for 1 and 4 h. The media was immediately collected, and cell lysates were stored at −80 °C until further analysis.

### Western blotting analysis

Following treatment, tissue culture plates of small luteal cells were immediately placed on ice and rinsed with 1 mL of ice-cold PBS three times to remove excess media. Cells were lysed in 75 µL of RIPA supplemented with 1 × protease and phosphatase inhibitors. Cells were removed from the culture dish using a cell scraper, and lysates were sonicated at 40% power setting (VibraCell, Model CV188) to ensure proper homogenization. Lysates were then centrifuged at 12,000 × *g* for 10 min at 4 °C, and the supernatant was collected. Protein concentrations were determined using the BCA protein assay kit. Samples were suspended in 6 × Laemmli buffer and placed in a dry heat bath at 90 °C for 6 min. Proteins (8–15 µg/sample) were resolved using 10% SDS-PAGE and electrophoretically transferred to nitrocellulose membranes. Membranes were blocked with 5% non-fat milk in Tris-buffered saline with 0.1% Tween-20 (TBS-T) for 1 h at room temperature. Membranes were incubated with primary antibodies (Table 1) at 4 °C overnight for the detection of total and phosphorylated proteins. The next day, membranes were rinsed three times with TBS-T for 5 min each, followed by incubation with the appropriate HRP-conjugated secondary antibody (Table 1) for 1 h at room temperature. Blots were washed with TBS-T, and SuperSignal West Femto Chemiluminescent Substrate was applied per the manufacturer’s instructions. Signals were visualized using the iBright Imaging System (Thermo Fisher Scientific), and the densitometry of immunoreactive proteins was determined using ImageJ software v1.52a. Protein levels were normalized to β-actin (ACTB). Fold changes in protein expression due to treatment were then determined.

**Table 1:** Characteristics of antibodies used for western blotting

Antibody name	Dilution ratio	Species specificity	Source	Supplier (distributor, town, country)	Cat. No	RRID
ACTB	1:5000	Bovine	Mouse mAb	Sigma Life Science (St. Louis, MO, USA)	A5441	AB_476744
HRP-linked Mouse	1:5000	Mouse	Secondary Ab	Jackson ImmunoResearch (West Grove, PA, USA)	115035205	AB_330924
HRP-linked Rabbit	1:5000	Rabbit	Secondary Ab	Jackson ImmunoResearch	111035003	AB_2099233
LATS1	1:1000	Mouse	Rabbit pAb	Cell Signaling (Boston, MA, USA)	3477S	AB_2133513
Phospho-ERK1/2 ^(Thr202/Tyr204)^	1:1000	Mouse	Rabbit pAb	Cell Signaling	9101L	AB_331646
Phospho-LATS1 ^(Ser909)^	1:1000	Mouse	Rabbit pAb	Cell Signaling	9157S	AB_2133515
Phospho-PKA substrates^(RRXS*/T*)^	1:1000	Mouse	Rabbit pAb	Cell Signaling	9624S	AB_331817
Phospho-TAZ ^(Ser89)^	1:1000	Mouse	Rabbit mAb	Cell Signaling	59971S	AB_2799578
Phospho-YAP1 ^(Ser127)^	1:1000	Mouse	Rabbit mAb	Cell Signaling	13008S	AB_2650553
Phospho-YAP1 ^(Ser397)^	1:1000	Mouse	Rabbit mAb	Cell Signaling	13619S	AB_2650554
STAR	1:2000	Mouse	Rabbit pAb	Abcam (Cambridge, UK)	ab96637	AB_10678397
TAZ	1:1000	Mouse	Rabbit mAb	Cell Signaling	8418S	AB_10950494
YAP1	1:1000	Mouse	Rabbit mAb	Cell Signaling	14074S	AB_2650491

*ACTB* β-actin (loading control), *mAb* monoclonal antibody, *pAb* polyclonal antibody, *HRP* horseradish peroxidase, *LATS1* Large Tumor Suppressor Kinase 1, *p-ERK1/2 (Thr202/Tyr204)* Phosphorylated Extracellular Signal-Regulated Kinases 1 and 2, Thr202/Tyr204, *p-LATS1 (Ser909)* Phosphorylated Large Tumor Suppressor Kinase 1, Ser909, *p-PKA substrates* Phosphorylated Protein Kinase A substrates, *p-TAZ (Ser89)* Phosphorylated TAZ (Transcriptional coactivator with PDZ-binding motif), Ser89, *p-YAP1 (Ser127)* Phosphorylated Yes-Associated Protein 1, Ser127, *p-YAP1 (Ser397)* Phosphorylated Yes-Associated Protein 1, Ser397, *STAR* Steroidogenic Acute Regulatory protein, *TAZ* (Transcriptional co-activator with PDZ-binding motif), *YAP1* Yes-Associated Protein 1

### Phos-tag SDS-PAGE analysis

Sample preparation was the same as in Western blotting; however, proteins were resolved using 8% Phos-tag SDS-PAGE. Resolving gels contained 8% acrylamide, 390 mM Tris–HCl (pH 8.8), 0.10% SDS, 20 µM Phos-tag acrylamide, and 100 µM MnCl₂, and were polymerized using APS and TEMED. Stacking gels were prepared under standard SDS-PAGE conditions without Phos-tag or MnCl₂. Following electrophoresis, gels were washed three times for 10 min in transfer buffer containing 10 mM EDTA, followed by a 10-min wash in transfer buffer without EDTA, then transferred by standard wet transfer and processed for immunoblotting.

### Progesterone analysis

Progesterone concentrations in culture media were determined using a commercially available enzyme-linked immunosorbent assay (ELISA) kit according to the manufacturer's protocol. Briefly, conditioned culture media samples were collected, and progesterone concentrations were quantified using the ELISA kit. All samples were assayed in duplicate to ensure accuracy and consistency in the measurements, with intra- and inter-assay coefficients of variation 7.03% and 11.4%, respectively.

### Cytoplasmic and nuclear isolation

Cytoplasmic and nuclear fractions were prepared from cultured small luteal cells (3 × 10⁶ cells per 10-cm dish) treated with or without LH for 1 h. Briefly, cells were washed with ice-cold PBS and scraped directly into ice-cold hypotonic lysis buffer (10 mM HEPES, 10 mM KCl, 1.5 mM MgCl₂, 0.1 mM EDTA; pH 7.5) supplemented with 1 × protease and phosphatase inhibitors and incubated for 15 min on ice. To monitor lysis efficiency, ~ 3 µL of the lysate was mixed at a 1:1 ratio with trypan blue and visualized with a microscope to confirm plasma membrane disruption and nuclear release. Lysates were centrifuged at 800 × *g* for 5 min at 4 °C to pellet nuclei. The supernatant was passed through a 20-µm filter to remove residual debris and centrifuged again at 800 × *g*. The clarified supernatant was then centrifuged at 14,000 × *g* for 10 min to obtain the cytoplasmic fraction. The nuclear pellet was washed once with hypotonic buffer, spun again at 800 × *g* for 5 min, and resuspended in RIPA buffer to extract nuclear proteins. Protein concentration was determined, and equal amounts of nuclear and cytoplasmic proteins (10 µg each) were analyzed by Western blotting.

### RNA isolation and sequencing

Total RNA was isolated from samples stored in TRIzol reagent according to the manufacturer’s instructions. Briefly, an equal volume of 100% ethanol was added to each TRIzol-lysed sample, and the mixture was loaded directly onto a Zymo-Spin™ IICR column. On-column, DNase I digestion was performed to eliminate residual genomic DNA. Following the recommended wash steps, RNA was eluted in 25 µL of DNase/RNase-free water. RNA concentration and purity were assessed using a NanoDrop™ 2000 spectrophotometer, and all samples were stored at −80 °C until further processing. RNA sequencing was performed by the University of Nebraska Medical Center Genomics Core Facility (RRID:SCR_023539). Purified RNA was used as input for library preparation with the Universal Plus mRNA-Seq with NuQuant Library Preparation Kit (Tecan), which includes poly(A) RNA selection, fragmentation, cDNA synthesis, and ligation of Illumina-compatible indexed adapters. Libraries were sequenced on an Illumina NovaSeq 6000 platform using 100-bp paired-end reads, with a target sequencing depth of approximately 40 million reads per sample.

### Bioinformatic analysis

Raw read pairs were processed using TrimGalore2 (RRID:SCR_011847) [18] to remove adapter sequences, low-quality reads (Phred score < 20), and reads shorter than 20 bp. Cleaned reads were aligned to the Bos taurus genome: ARS-UCD2.0 and annotated using STAR [19]. Gene-level quantification was also performed in STAR. Differential expression analysis was conducted in RStudio using the DESeq2 package [20]. Genes with fewer than 10 counts in the number of samples in the smallest treatment group were filtered out prior to analysis. The statistical design included treatment as the primary factor and sample ID as a blocking factor. Significance thresholds were set at FDR-adjusted *p* < 0.05 and |log2 fold change|> 1.0. Significantly differentially expressed genes were analyzed using Ingenuity Pathway Analysis (IPA) to identify upstream regulators. Regulators with a z-score ≥|2| and *p* < 0.05 were considered significantly affected.

### YAP1 and TAZ knockdown

Cells were transfected with siCTL, siYAP1, or siTAZ for 6 h using Lipofectamine RNAiMAX in Opti-MEM media. Following incubation, an equal volume of 2 × M199 (supplemented with 10% FBS and 2 × penicillin–streptomycin) was added, yielding a final concentration of 1 × M199 containing 5% FBS and 1 × penicillin- streptomycin. To ensure efficient knockdown, a second transfection was performed 24 h later. Cells were maintained at 37 °C in a 95% humidified atmosphere with 5% CO₂ throughout the 48 h knockdown period before hormone treatment and further analyses. For progesterone, the media was replaced with serum free M199 and cells were equilibrated for 2 h before treatment with control (M199 medium) or LH (10 ng/mL) for 4 h. Following treatment, conditioned media was collected to measure progesterone concentrations, and cell lysates were harvested for Western blot analysis to confirm successful knockdown of YAP1 and TAZ.

### YAP1 and TAZ overexpression

Overexpression of constitutively active YAP1 and TAZ were achieved using adenoviral vectors expressing either mutant YAP1^S127A^ or mutant TAZ^S89A^. The adenoviral constructs encoding mutant YAP1 (Ad. YAP1^S127A^) and TAZ (Ad. TAZ^S89A^) were prepared as previously described [5]. Briefly, enriched small and large luteal cells were seeded into culture dishes for 24 h prior to infection with the adenoviruses. The Ad. YAP1^S127A^ or Ad. TAZ^S89A^ viruses were added to the cultures in serum-free M199 medium. After 2 h of infection, the media was replaced with M199 enriched with 10% FBS and 1 × Penicillin Streptomycin Solution, and the cultures were maintained for an additional 48 h. For progesterone analysis, the media was replaced with serum free M199 and cells were equilibrated for 2 h before treatment with control (M199 medium) or LH (10 ng/mL) for 4 h. Following treatment, conditioned media was collected to measure progesterone concentrations, and cell lysates were harvested for Western blot analysis to confirm overexpression of mutant YAP1 or TAZ.

### Statistical analysis

Each experiment was performed at least three times using follicular (granulosa and theca) and luteal (small and large) cells from separate animals. Data are presented as mean ± standard error of the mean (SEM). Statistical analyses were conducted using t-test, one- or two-way ANOVA as appropriate, followed by post hoc tests indicated in each figure legend. A *p*-value of < 0.05 was considered statistically significant. All graphical analyses were generated using GraphPad PRISM (version 10.4).

## Results

### Hippo signaling components increase during in vitro differentiation of bovine theca and granulosa cells

**Fig. 1:**
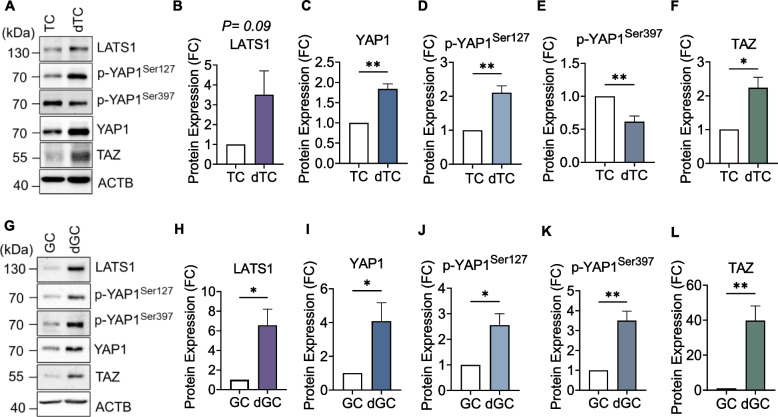
In vitro differentiation of theca and granulosa cells upregulates Hippo pathway components. Bovine theca and granulosa cells were cultured under differentiation conditions for 96 h. **A**, **G** Representative immunoblots of LATS1, p-YAP1^Ser127^, p-YAP1^Ser397^, YAP1, and TAZ in undifferentiated (TC, GC) and differentiated theca and granulosa cells (dTC, dGC). **B**-**F** Quantification of protein expression from (A), shown as fold change (FC). **H**–**L** Quantification of protein expression from (G), shown as fold change (FC). ACTB served as a loading control. Data means are ± SEM (*n* = 6 independent experiments). Paired t-test: *P < 0.05, **P < 0.01. Abbreviations: LATS1, large tumor suppressor kinase 1; p-YAP1^Ser127/397^, phosphorylated Yes-associated protein 1 at Serine 127/397; YAP1, Yes-associated protein 1; TAZ, transcriptional co-activator with PDZ-binding motif; ACTB, β-actin

To assess changes in Hippo signaling proteins during differentiation, theca and granulosa cells were cultured under established conditions to induce differentiation. Immunoblotting showed higher LATS1, YAP1 p-YAP1^Ser127^ and TAZ, in differentiated theca and granulosa cells compared to undifferentiated (Fig. 1A, G). Relative to undifferentiated cells, differentiated theca cells exhibited increases in YAP1 (1.8-fold), p-YAP1^Ser127^ (2.1-fold), and TAZ (2.2-fold), along with a decrease in p-YAP1^Ser397^ (0.61-fold) (Fig. 1C-F; *p* < 0.05), whereas the increase in LATS1 (3.5-fold) did not reach statistical significance (Fig. 1B; *p* = 0.09). Differentiated granulosa cells displayed greater increases in Hippo signaling protein during differentiation, including increased LATS1 (6.5-fold), YAP1 (4.0-fold), p-YAP1^Ser127^ (2.5-fold), p-YAP1^Ser397^ (3.5-fold), and TAZ (39.7-fold), all of which were statistically significant (Fig. 1H-L; *p* < 0.05). Thus, differentiation of follicular cells is associated with increased abundance and phosphorylation of Hippo pathway components, with the exception of reduced p-YAP1^Ser397^ in differentiated theca cells (Supplementary Fig. 1A-D).

### Luteinizing hormone (LH) acutely activates Hippo signaling in bovine small luteal cells

**Fig. 2:**
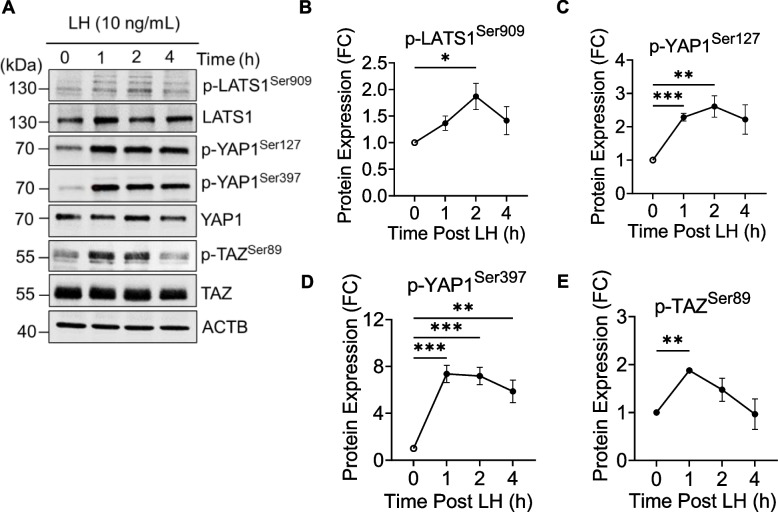
LH stimulates activation of the Hippo pathway. Bovine small luteal cells were treated with LH (10 ng/mL) for up to 4 h. **A** Representative immunoblots of phosphorylated and total LATS1, YAP1, and TAZ. ACTB serves as a loading control. **B**-**E** Quantification of phosphorylated LATS1^Ser909^, p-YAP1^Ser127^, p-YAP1^Ser397^, and TAZ^Ser89^, expressed as fold change (FC); LH vs. 0 h. Data means are ± SEM (*n* = 3–6 independent experiments). One-way ANOVA with Dunnett’s multiple comparisons: *P < 0.05, **P < 0.01, ***P < 0.001. Abbreviations: LH, luteinizing hormone; p-LATS1.^Ser909^, phosphorylated LATS1 at Serine 909

To determine whether LH regulates Hippo signaling in luteal cells, small luteal cells were stimulated with LH (10 ng/mL) for up to 4 h. Western blot analysis revealed a rapid and transient increase in phosphorylation of multiple Hippo pathway components following LH treatment (Fig. 2A). At 1 h, LH stimulation increased p-YAP1^Ser127^ (2.2-fold), p-YAP1^Ser397^ (7.3-fold), and p-TAZ^Ser89^ (1.8-fold), and at 2 h p-LATS1^Ser909^ peaked at 2 h (1.8-fold), (Fig. 2B-E; *p* < 0.05). These kinetics indicate acute activation of the Hippo cascade by LH. Consistent with these findings, Phos-tag SDS-PAGE revealed a clear mobility shift of YAP1 following LH and forskolin (FSK; an activator of adenylyl cyclase that increases cAMP) treatment, indicative of increased phosphorylation (Supplementary Fig. 2).

### LH-induced activation of Hippo signaling pathway promotes cytoplasmic retention of YAP1 and TAZ

**Fig. 3:**
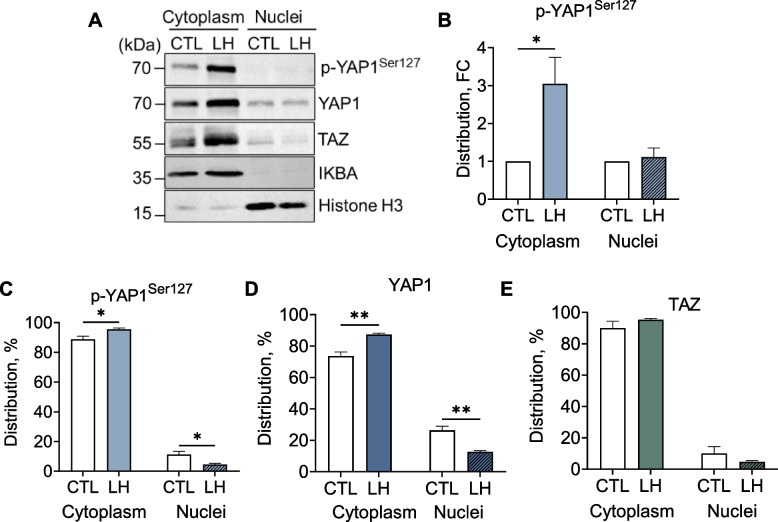
LH induces YAP1/TAZ retention in the cytoplasm. Bovine small luteal cells were treated with or without LH (10 ng/mL) for 1 h, followed by subcellular fractionation. **A** Representative immunoblots showing p-YAP1^Ser127^, total YAP1 and TAZ in cytoplasmic and nuclear fractions. IKBA (IκBα) and Histone H3 were used as cytoplasmic and nuclear markers, respectively. **B**, **C** The graphs show FC and percentage distribution of cytoplasmic and nuclear p-YAP1^Ser127^, following LH treatment. **D**, **E** The graphs show total YAP1 and TAZ distribution percentages in cytoplasmic and nuclear fractions, following LH treatment. Data means are ± SEM (*n* = 3 independent experiments). Two-way ANOVA with uncorrected Fisher’s LSD: *P < 0.05, **P < 0.01. Abbreviations: IKBA (IκBα), inhibitor of nuclear factor kappa B alpha

To determine whether LH alters the subcellular localization of Hippo pathway effectors, small luteal cells were treated with LH (10 ng/mL) for 1 h and fractionated into cytoplasmic and nuclear pools. Under basal conditions, YAP1 and TAZ were present in both cytoplasmic and nuclear fractions; LH stimulation shifted their distribution toward the cytoplasm (Fig. 3A). Quantitative analysis revealed that cytoplasmic p-YAP1^Ser127^ increased by ~ threefold in LH-treated cells compared to control cells (Fig. 3B; *p* < 0.05) and was detected predominantly in the cytoplasmic fraction (Fig. 3C; *p* < 0.05). Total YAP1 exhibited a significant redistribution from the nucleus to the cytoplasm following LH stimulation, characterized by increased cytoplasmic abundance and a concomitant reduction in nuclear YAP1 (Fig. 3D; *p* < 0.01, and Supplementary Fig. 3 A, B).TAZ localization followed a similar overall pattern, with signal predominantly detected in the cytoplasmic fraction and low nuclear abundance across conditions (Fig. 3E). Fraction purity was confirmed by IKBA (cytosolic) and Histone H3 (nuclear) markers. Thus, LH-induced Hippo activation promotes phosphorylation-dependent cytoplasmic retention and nuclear exclusion of YAP1 and TAZ in bovine small luteal cells.

### RNA-Seq identifies YAP1/TAZ as inhibited upstream regulators following LH stimulation

Small luteal cells were treated with LH for 4 h and subjected to RNA-Seq analysis. Upstream regulator analysis using Ingenuity Pathway Analysis (IPA) predicted significant inhibition of YAP1, TAZ and their transcriptional partner, TEADs, at the 4 h time point (Table 2). Accordingly, transcription of Hippo pathway components and canonical YAP1/TAZ target genes were downregulated in LH-treated cells (Supplementary Fig. 4A-I). As expected, STAR expression increased robustly at 4 h in response to LH (Supplementary Fig. 4 F), supporting progesterone production.

**Table 2:** Predicted upstream regulators: 4 vs 0 h

Upstream Regulator	Full Name	# of Targeted Molecules	z-score	*P*-Value
Activation				
LH (complex)	Luteinizing Hormone	25	3.309	1.75E-20
PKA (complex)	Protein Kinase A	24	3.627	7.39E-11
SREBF1	Sterol Regulatory Element-Binding Transcription Factor 1	23	2.328	1.70E-05
Inhibition				
WWTR1/TAZ	WW domain containing transcription regulator 1	12	−2.962	3.63E-03
YAP1/TAZ	Yes-Associated Protein 1	5	−2.207	8.69E-03
TEAD3	TEA Domain Transcription Factor 3	8	−2.135	7.41E-05
TEAD1	TEA Domain Transcription Factor 1	10	−1.941	1.80E-02

### LH-induced phosphorylation of YAP1 requires PKA and LATS1/2, but not ERK1/2 activity

**Fig. 4:**
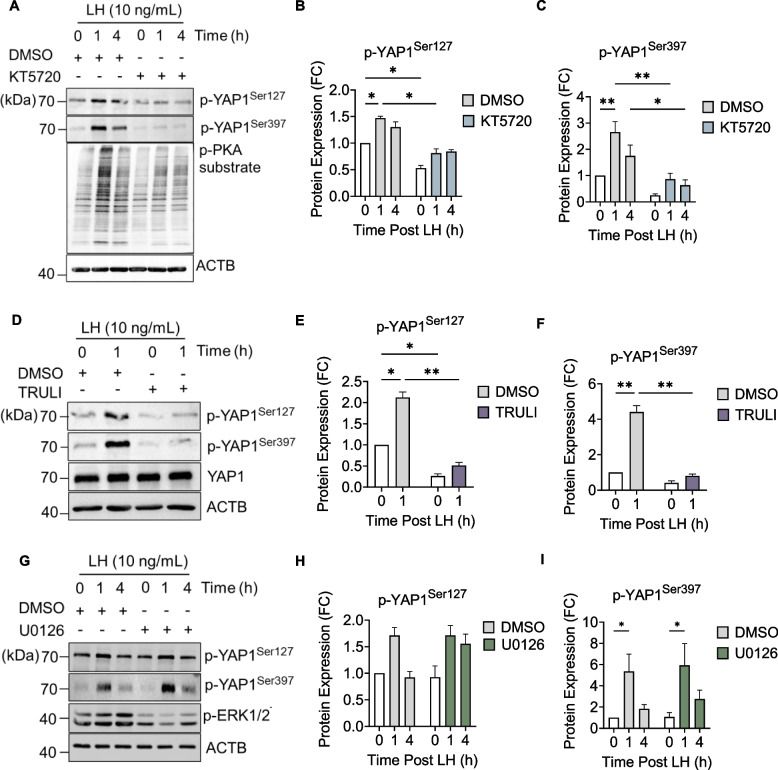
LH stimulates YAP1 phosphorylation via PKA and LATS1/2, independent of ERK1/2. Bovine small luteal cells were pre-treated with KT5720 (PKA inhibitor, 10 µM), U0126 (MEK1/2 inhibitor, 10 µM), and TRULI (LATS1/2 inhibitor, 10 µM), then stimulated with LH (10 ng/mL) for 0, 1, or 4 h. **A** Representative immunoblot showing LH-induced phosphorylation of YAP1^Ser127^ and YAP1^Ser397^ and PKA substrate phosphorylation ± KT5720. **B**, **C** Quantification of p-YAP1^Ser127^ and p-YAP1^Ser397^ expression normalized to 0 h. **D** Representative immunoblot showing LH-induced phosphorylation of YAP1^Ser127^ and YAP1^Ser397^ ± TRULI. **E**, **F** Quantification of p-YAP1^Ser127^ and p-YAP1^Ser397^ expression normalized to 0 h. **G** Representative immunoblot showing LH-induced phosphorylation of YAP1^Ser127^, and YAP1^Ser397^ and ERK1/2^Thr202/Tyr204^ ± U0126. **H**, **I** Quantification of p-YAP1^Ser127^ and p-YAP1^Ser397^ expression normalized to 0 h. Data represent mean ± SEM (*n* = 3–4 independent experiments). Two-way ANOVA with Tukey’s multiple comparisons: *P < 0.05, **P < 0.01. Abbreviations: PKA, protein kinase A; ERK1/2, extracellular signal-regulated kinases 1 and 2; MEK1/2, mitogen-activated protein kinases 1 and 2; p-ERK1/2.^Thr202/Tyr204^, phosphorylated ERK1/2 at Threonine 202 and Tyrosine 204

To define the upstream signaling events mediating LH-induced activation of Hippo pathway, pharmacological inhibitors targeting canonical LH signaling via PKA, core Hippo kinases LATS1/2, and ERK1/2 [21], were employed. Small luteal cells were pretreated for 1 h with KT5720 (PKA inhibitor), TRULI (LATS1/2 inhibitor), or U0126 (MEK1/2 inhibitor), followed by LH stimulation (10 ng/mL) for up to 4 h. KT5720 abolished LH-induced increase in p-YAP1^Ser127^ and p-YAP1^Ser397^, and reduced global PKA-substrate phosphorylation to basal levels (Fig. 4A-C; *p* < 0.05). Inhibition of LATS1/2 with TRULI similarly suppressed LH-induced YAP1 phosphorylation at both Ser127 and Ser397 to near baseline (Fig. 4D-F; *p* < 0.05), without significantly altering progesterone production (Supplementary Fig. 5). U0126 effectively reduced p-ERK1/2^Thr202/Tyr204^ but did not affect p-YAP1^Ser127^ or p-YAP1^Ser397^ (Fig. 4G-I). Together, these data place PKA and LATS1/2 upstream of YAP1 phosphorylation in response to LH, independent of ERK1/2.

### Hippo signaling modulates progesterone production in bovine small luteal cells

**Fig. 5:**
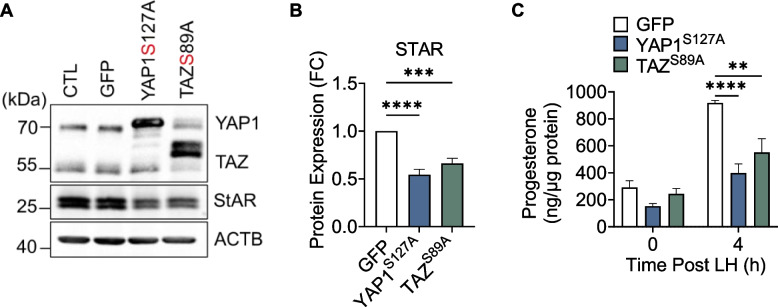
Constitutively active YAP1 and TAZ mutants suppress LH-induced progesterone production. Bovine small luteal cells were infected with adenoviruses (Ad) overexpressing GFP (control), constitutively active YAP1^S127A^ or constitutively active TAZ^S89A^ for 48 h and stimulated with LH (10 ng/mL) for 0 or 4 h. **A** Representative immunoblot showing overexpression of constitutively active YAP1^S127A^ and TAZ^S89A^ compared to GFP control in small luteal cells. ACTB was used as a loading control. **B** Quantification of STAR protein expression in cells expressing GFP, YAP1S127A, or TAZS89A, normalized to ACTB and expressed relative to GFP control. **C** Progesterone levels measured by ELISA in conditioned media from cells expressing GFP, YAP1^S127A^, or TAZ^S89A^ treated with LH. Data represent mean ± SEM (*n* = 3–6 independent experiments). Two-way ANOVA with Tukey’s multiple comparisons: **P < 0.01, ***P < 0.001, ****P < 0.0001. Abbreviations: YAP1^S127A^, constitutively active mutant of YAP1 with Serine 127 substituted by alanine; TAZ^S89A^, constitutively active mutant of TAZ with Serine 89 substituted by alanine; STAR, Steroidogenic Acute Regulatory protein

To determine the functional role of Hippo pathway effectors, YAP1 and TAZ, in progesterone synthesis, small and large luteal cells were infected with adenoviruses encoding constitutively active mutants of YAP1 (YAP1^S127A^) or TAZ (TAZ^S89A^), which are resistant to inhibitory phosphorylation and thus, remain transcriptionally active (Fig. 5A) [22]. Following overexpression, small luteal cells were stimulated with or without LH for 4 h. Constitutively active YAP1S127A or TAZS89A reduced STAR protein expression by ~ twofold (Fig. 5 B; *p* < 0.001). In parallel, LH-induced progesterone production also decreased by 54.4% ± 10.5% and 34.2% ± 13.9% in cells expressing YAP1S127A and TAZS89A, respectively (Fig. 5C; *p* < 0.01). In large luteal cells, progesterone levels measured under basal conditions were likewise significantly reduced following overexpression of constitutively active mutants of YAP1 or TAZ (Supporting Fig. 6; *p* < 0. 01).

**Fig. 6:**
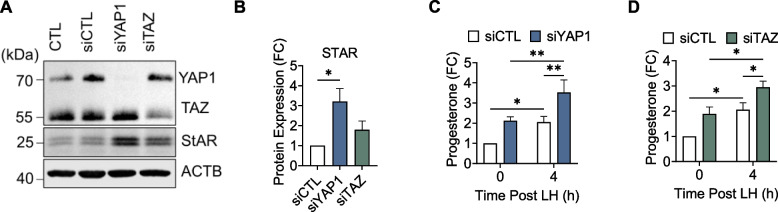
YAP1/TAZ knockdown enhances progesterone production. Bovine small luteal cells were transfected with siCTL, siYAP1, or siTAZ for 48 h and stimulated with LH (10 ng/mL) for 0 or 4 h. **A** Representative immunoblot confirming YAP1 and TAZ knockdown by siRNAs; ACTB, loading control. **B** Quantification of STAR protein expression following siYAP1 or siTAZ knockdown, normalized to ACTB and expressed relative to siCTL. **C**, **D** Progesterone levels at 0 and 4 h post-LH in cells transfected with siYAP1 (B) or siTAZ (C) compared to siCTL. Data represent mean ± SEM (*n* = 3 independent experiments). Two-way ANOVA with uncorrected Fisher’s LSD: *P < 0.05, **P < 0.01. Abbreviations: siCTL, small interfering RNA control; siYAP1, small interfering RNA targeting YAP1; siTAZ, small interfering RNA targeting TAZ

To further evaluate the role of YAP1 and TAZ in LH-induced steroidogenesis, small luteal cells were transfected with siRNAs targeting YAP1 or TAZ, resulting in knockdown efficiencies of 93.1% ± 2.7% for YAP1 and 59.4% ± 8.9% for TAZ, confirmed by Western blotting (Fig. 6A and Supporting Fig. 7). Functionally, this knockdown increased STAR protein expression by ~ threefold following suppression of YAP1 (Fig. 6B; *p* < 0. 05); and showed an increasing trend of ~ 1.8-fold with siTAZ-induced suppression of TAZ (Fig. 6B; *p* = 0.41). Consistent with this finding, LH-induced progesterone synthesis was enhanced by 71.5% ± 30.3% and 43.3% ± 11.9% for YAP1 and TAZ knockdown, respectively (Fig. 6C, D; *p* < 0.05).Together, these gain- and loss-of-function data indicate that YAP1/TAZ act as brakes on LH-stimulated steroidogenesis.

## Discussion

The present study identifies a previously unappreciated role for the Hippo signaling pathway in regulating LH-mediated progesterone synthesis in bovine small luteal cells. LH stimulation activates Hippo signaling, resulting in increased phosphorylation and cytoplasmic retention of the transcriptional co-activators YAP1 and TAZ. This LH-driven activation, largely mediated through the cAMP/PKA/LATS1/2 axis, dampens YAP1/TAZ transcriptional activity and coincides with robust progesterone production. These findings point to a distinct function for Hippo signaling in terminally differentiated, non-proliferative luteal cells, where limiting YAP1/TAZ activity appears to be permissive for maximal progesterone synthesis.

During luteinization, increased abundance and phosphorylation of Hippo pathway components suggest that this pathway is engaged as early as cells acquire a progesterone-dominant phenotype. Importantly, our study focuses on small luteal cells, which are highly LH-responsive. LH stimulation further amplifies Hippo signaling through the cAMP/PKA/LATS1/2 axis, promoting phosphorylation-dependent cytoplasmic sequestration of YAP1 and TAZ and reducing their nuclear co-activator activity. Consistent with this model, gain- and loss-of-function approaches show that expression of exogenous transcriptionally active YAP1/TAZ mutants suppress LH-induced progesterone production in small luteal cells. Large luteal cells were included only to assess YAP1/TAZ function under basal, LH-independent conditions, given their constitutive cAMP/PKA activity and high progesterone production despite limited responsiveness to acute LH [23]. In this context, YAP1/TAZ activation suppressed basal progesterone synthesis in large luteal cells. Furthermore, genetic repression of YAP1 or TAZ, through siRNA-mediated knockdown, enhances LH-induced steroidogenesis in small luteal cells. Although the downstream mechanisms remain to be fully resolved, the changes in STAR abundance when YAP1 and TAZ were either overexpressed or knocked down suggest that YAP1/TAZ work at the level of cholesterol transport [24]. In this way, Hippo signaling may help coordinate constraints on transcriptional output with the metabolic demands of progesterone synthesis during luteal activation.

This regulatory behavior is consistent with a broader body of work across endocrine tissues showing that YAP1 and TAZ modulate steroidogenic capacity, often acting as a transcriptional brake whose activity must be carefully controlled to permit hormone production [9, 25]. In Leydig cells, which are LH-responsive and share functional parallels with small luteal cells, TAZ suppresses steroidogenic enzyme expression through NR4A1 (Nuclear Receptor 4A1), and loss of TAZ enhances testosterone synthesis [14]. Similarly, repression of YAP1/TAZ downstream of FSH signaling in rat granulosa cells promotes expression of key steroidogenic enzymes, including CYP19A1, CYP11A1, and HSD3B, supporting estrogen synthesis [13, 26]. Consistent with these findings, constitutive activation of YAP1 in primary human granulosa cells reduces basal and forskolin (FSK)-stimulated estrogen and progesterone production and suppresses CYP19A1 and CYP11A1 expression, further supporting an inhibitory role for YAP1 in steroidogenesis [27]. In contrast, YAP1 knockdown in adult granulosa cell tumors compromises aromatase expression and leads to reduced estrogen production [12], underscoring the context-dependent role of YAP1 in granulosa cell steroidogenesis.

Luteal cells differ fundamentally from other steroidogenic cell types. Unlike proliferative or tumor-derived granulosa cells, luteal cells are terminally differentiated and non-proliferative [28], suggesting that Hippo signaling in this setting is uncoupled from growth control and instead repurposed to regulate biosynthetic output. Importantly, the role of Hippo signaling in luteal progesterone synthesis appears to operate on distinct temporal scales, with acute pathway activation being permissive for LH action, while sustained YAP1/TAZ activity exerts longer-term constraints on steroidogenic capacity. Although LH acutely activates the cAMP/PKA/LATS1/2 axis and promotes YAP1/TAZ phosphorylation and cytoplasmic sequestration, this short-term modulation alone does not directly alter progesterone output, as acute inhibition of LATS1/2, by TRULI inhibitor, has no effect on LH-stimulated progesterone synthesis. This indicates that Hippo pathway activation is permissive rather than instructive for acute LH-driven progesterone synthesis, which proceeds independently of YAP1/TAZ phosphorylation status on this timescale. By contrast, gain- and loss-of-function experiments indicate that sustained YAP1/TAZ activity constrains steroidogenic capacity, with transcriptionally active YAP1/TAZ suppressing progesterone production, and their depletion enhancing LH-induced steroidogenesis. These temporal distinctions suggest that YAP1 and TAZ influence steroidogenesis by shaping the cellular state that supports hormone production such as differentiation state or gonadotropin responsiveness [27], rather than by acting as immediate regulators of progesterone output.

Consistent with this interpretation, transcriptomic profiling following LH stimulation identifies YAP1, TAZ and TEAD family members as inhibited upstream regulators at 4 h, supporting repression of YAP1/TEAD-dependent transcriptional programs downstream of LH. Support for this state-based model also comes from other systems in which Hippo signaling is linked to metabolic remodeling independent of acute growth effects [29]. In hepatocytes, YAP1 interacts with SREBP1-c and SREBP2 transcription factors to promote lipogenesis and cholesterol biosynthesis, thereby increasing lipid substrate availability rather than directly controlling downstream metabolic flux [11]. By analogy, YAP1/TAZ in differentiated luteal cells may similarly influence progesterone synthesis by modulating cholesterol handling and availability rather than growth or proliferation. Consistent with this interpretation, changes in STAR protein abundance following manipulation of YAP1/TAZ activity point to altered cholesterol transport, a rate-limiting step in progesterone synthesis. Together, these observations support a framework in which acute LH signaling engages Hippo pathway components, while longer-term YAP1/TAZ activity establishes the cellular state that enables maximal progesterone synthesis during luteal activation.

Meanwhile this study establishes YAP1 and TAZ as transcriptional restraints on LH-stimulated progesterone synthesis in luteal cells, several mechanistic questions remain open. It is not yet clear whether YAP1/TAZ directly regulate the genes involved in steroidogenesis, cholesterol trafficking, or whether their effects are mediated through intermediary transcription factors or metabolic signaling pathways. ChIP-seq or CUT&RUN approaches targeting YAP1/TAZ and TEAD factors in luteal cells could identify direct transcriptional targets and reveal whether steroidogenic or cholesterol trafficking genes are under their immediate control. In addition, although LH represses canonical YAP1/TAZ activity, the broader transcriptional landscape associated with Hippo pathway modulation in luteal cells remains to be defined and will require more comprehensive transcriptomic analyses. Time-resolved RNA-seq following acute LATS1/2 inhibition or YAP1/TAZ depletion, combined with chromatin accessibility profiling, would help distinguish direct from indirect transcriptional effects and clarify the kinetics of pathway engagement. Finally, potential interactions between Hippo signaling and other metabolic regulators of steroidogenesis, including mTOR (mechanistic Target of Rapamycin) [30] and AMPK (AMP-activated Protein Kinase) [1, 31] represent layers of regulation that merit further investigation. Targeted pathway inhibition studies could define how Hippo signaling interacts with other metabolic regulators to control progesterone synthesis.

### Limitations

Our study provides novel insights into the role of Hippo signaling in luteal steroidogenesis; however, certain limitations should be acknowledged. First, the use of an in vitro culture system may not fully replicate the complexity of the in vivo luteal environment; therefore, future in vivo studies will be necessary to validate the physiological relevance of our findings. Second, our work primarily focused on small luteal cells without a comprehensive evaluation of large luteal cell populations, which may exhibit distinct regulatory mechanisms. Third, these experiments primarily focused on acute responses to LH in mid-cycle small luteal cells, and the dynamics of Hippo signaling across luteal lifespan (early, mid, late, regressing stages) were not addressed, leaving questions about stage-specific regulation. In addition, while acute LH treatments were used to examine rapid Hippo pathway signaling dynamics, YAP1/TAZ knockdown and overexpression approaches reflect longer-term modulation of the pathway activity and may engage compensatory mechanisms that differ from immediate LH-driven effects. Lastly, although we demonstrated regulation of YAP1/TAZ phosphorylation and localization in relation to progesterone production, additional layers of Hippo pathway regulation and their broader metabolic impacts on luteal function remain to be explored.

## Conclusion

This study identifies a previously unrecognized role for Hippo signaling in shaping LH-mediated progesterone synthesis in the corpus luteum. Through activation of the cAMP/PKA pathway, LH suppresses YAP1 and TAZ transcriptional activity, suggesting that Hippo signaling limits steroidogenic capacity rather than acting as an acute driver of progesterone synthesis. In terminally differentiated luteal cells, this pathway appears repurposed from its canonical role in growth control to support the biosynthetic demands of progesterone synthesis, in part by coordinating transcriptional programs with cholesterol handling and metabolic state. Together, these findings expand current models of luteal regulation by integrating Hippo signaling into the molecular framework governing progesterone production and luteal function. Disruption of this regulatory balance may contribute to impaired luteal competence, including luteal phase deficiency or early pregnancy loss. Future studies are needed to define how hormonal and mechanical cues converge on Hippo signaling in the ovary and to determine whether modulation of this pathway can be leveraged to improve luteal function and reproductive outcomes.

## Supplementary Information

Supplementary Material 1: Supporting Figure 1. Ratio of p-YAP1^Ser127^ and p-YAP1^Ser397^ to total YAP1 in differentiated TC and GC. Bovine theca and granulosa cells were cultured under luteinizing conditions for 96 h.Quantified ratio of p-YAP1^Ser127^ and p-YAP1^Ser3^97 to total YAP1 protein expression shown as fold changein differentiated TC and differentiated GC, respectively. ACTB, loading control. Data means are ± SEM. Paired t-test: ****P < 0.0001.

Supplementary Material 2: Supporting Figure 2. The shift in phosphorylation of YAP1 upon LH/FSK treatment. Bovine small luteal cells were treated with LHor forskolinfor 30 minutes. Representative Phos-tag SDS-PAGE showing a mobility shift of YAP1 in response to LH and FSK, indicative of increased phosphorylation.

Supplementary Material 3: Supporting Figure 3. Subcellular localization of Hippo effectors. Bovine small luteal cells were treated with or without LHfor 1 h, followed by subcellular fractionation.The graphs present total YAP1 and TAZ FC distribution in cytoplasmic and nuclear fractions, following LH treatment.

Supplementary Material 4: Supporting Figure 4. Downregulation of YAP1/TAZ target genes following LH-induced inhibition. Bovine small luteal cells were treated with LHfor 0 or 4 h, followed by RNA-seq.Representative graphs show downregulation of Hippo pathway components andwell-known YAP1 and TAZ target genes.Upregulation of STAR. Data means are ± SEM. Paired t-test: *P < 0.05, **P < 0.01, ***P < 0.001.

Supplementary Material 5: Supporting Figure 5. LATS1/2 inhibition by TRULI showed no significant change in progesterone output. Bovine small luteal cells were pre-treated for 1 h with TRULI, then stimulated with or without LHfor 1 h. Progesterone levels measured by ELISA. Data represent mean ± SEM. Two-way ANOVA with uncorrected Fisher’s LSD: *P < 0.05.

Supplementary Material 6: Supporting Figure 6. Constitutively active mutant YAP1 and TAZ suppress basal progesterone production in large luteal cells. Bovine large luteal cells were infected with adenovirusesoverexpressing GFP, constitutively active YAP1^S127A^ or constitutively active TAZ^S89A^ for 48 h. Progesterone levels measured by ELISA. Data represent mean ± SEM. One-way ANOVA with Dunnett’s multiple comparisons: **P < 0.01, ****P < 0.0001.

Supplementary Material 7: Supporting Figure 7. Successful Si-mediated YAP1/TAZ knockdown. Bovine small luteal cells were transfected with siRNAs targeting YAP1 or TAZ.YAP1 and TAZ Knockdown efficiencies were 93.1% ± 2.7% and 59.4% ± 8.9%, respectively. ACTB, loading control. Data means are ± SEM. Paired t-test: *P < 0.05, ***P < 0.001.

## Data Availability

All data generated or analyzed in this study are included in this published article or are available upon reasonable request from the authors.

## Abbreviations

Ad. TAZS89A: Adenovirus encoding mutant TAZ with serine 89 to alanine substitution
Ad. YAP1S127A: Adenovirus encoding mutant YAP1 with serine 127 to alanine substitution
BCA: Bicinchoninic acid (protein assay)
BSA: Bovine serum albumin
cAMP: Cyclic adenosine monophosphate
CYP19A1: Cytochrome P450 Family 19 Subfamily A Member 1
CYP11A1: Cytochrome P450 Family 11 Subfamily A Member 1
dTC: Differentiated Theca cells
dGC: Differentiated Granulosa cells
DMEM: Dulbecco’s Modified Eagle’s Medium
EDTA: Ethylenediaminetetraacetic acid
EGTA: Ethylene glycol-bis (β-aminoethyl ether)-N, N, N′, N′-tetraacetic acid
ELISA: Enzyme-linked immunosorbent assay
ERK1/2: Extracellular Signal-Regulated Kinase 1/2
FASN: Fatty Acid Synthase
FBS: Fetal bovine serum
FSK: Forskolin
HMGCR: 3-Hydroxy-3-Methylglutaryl-CoA Reductase
HRP: Horseradish peroxidase
HSD3B: 3β-Hydroxysteroid Dehydrogenase
KGN: Human granulosa-like tumor
LATS1/2: Large Tumor Suppressor Kinases 1 and 2
LH: Luteinizing hormone
MEK1/2: Mitogen-Activated Protein Kinases 1 and 2
MST1/2: Mammalian STE20-like Protein Kinases 1 and 2
p-LATS1 (Ser909): Phosphorylated LATS1 at serine 909
p-TAZ (Ser89): Phosphorylated TAZ at serine 89
p-YAP (Ser127/Ser397): Phosphorylated YAP1 at serine 127 or serine 397
PKA: Protein Kinase A
PMA: Phorbol myristate acetate
PGF2α: Prostaglandin F2 alpha
RUNX: Runt-Related Transcription Factors
SDS-PAGE: Sodium Dodecyl Sulfate–Polyacrylamide Gel Electrophoresis
siLATS1: SiRNA targeting LATS1
siRED: Non-targeting siRNA control
siRNA: Small interfering RNA
siTAZ: SiRNA targeting TAZ
siYAP1: SiRNA targeting YAP1
SMADs: Mothers Against Decapentaplegic Homologs (involved in TGF-β signaling)
SREBF1: Sterol Regulatory Element Binding Transcription Factor 1
STAR: Steroidogenic Acute Regulatory Protein
TAZ: Transcriptional Co-Activator with PDZ-Binding Motif (also known as WWTR1)
TBS-T: Tris-buffered saline with Tween-20
TEADs: TEA Domain Transcription Factors 1–4
YAP1: Yes-Associated Protein 1

## References

[1] Przygrodzka E, Plewes MR, Davis JS. Luteinizing hormone regulation of inter-organelle communication and fate of the corpus luteum. Int J Mol Sci. 2021;22(18):9972. 34576135 10.3390/ijms22189972 PMC8470545

[2] Stocco C, Telleria C, Gibori G. The molecular control of corpus luteum formation, function, and regression. Endocr Rev. 2007;28(1):117–49. 17077191 10.1210/er.2006-0022

[3] Fitz TA, Mayan MH, Sawyer HR, Niswender GD. Characterization of two steroidogenic cell types in the ovine corpus luteum1, 2. Biol Reprod. 1982;27(3):703–11. 6291651 10.1095/biolreprod27.3.703

[4] Niswender GD, Juengel JL, Silva PJ, Rollyson MK, McIntush EW. Mechanisms controlling the function and life span of the corpus luteum. Physiol Rev. 2000;80(1):1–29. 10617764 10.1152/physrev.2000.80.1.1

[5] Plewes MR, Krause C, Talbott HA, Przygrodzka E, Wood JR, Cupp AS, et al. Trafficking of cholesterol from lipid droplets to mitochondria in bovine luteal cells: Acute control of progesterone synthesis. FASEB J. 2020;34(8):10731–50. 32614098 10.1096/fj.202000671R PMC7868007

[6] Yu FX, Zhao B, Guan KL. Hippo pathway in organ size control, tissue homeostasis, and cancer. Cell. 2015;163(4):811–28. 26544935 10.1016/j.cell.2015.10.044 PMC4638384

[7] Kwon H, Kim J, Jho EH. Role of the Hippo pathway and mechanisms for controlling cellular localization of YAP/TAZ. FASEB J. 2022;289(19):5798–818. 10.1111/febs.16091 34173335

[8] Zhao B, Ye X, Yu J, Li L, Li W, Li S, et al. TEAD mediates YAP-dependent gene induction and growth control. Genes Dev. 2008;22(14):1962–71. 18579750 10.1101/gad.1664408 PMC2492741

[9] Clark KL, George JW, Przygrodzka E, Plewes MR, Hua G, Wang C, et al. Hippo signaling in the ovary: emerging roles in development, fertility, and disease. Endocr Rev. 2022;43(6):1074–96. 35596657 10.1210/endrev/bnac013 PMC9695108

[10] Ibar C, Irvine KD. Integration of Hippo-YAP signaling with metabolism. Dev Cell. 2020;54(2):256–67. 32693058 10.1016/j.devcel.2020.06.025 PMC7373816

[11] Shu Z, Gao Y, Zhang G, Zhou Y, Cao J, Wan D, et al. A functional interaction between Hippo-YAP signalling and SREBPs mediates hepatic steatosis in diabetic mice. J Cell Mol Med. 2019;23(5):3616–28. 30821074 10.1111/jcmm.14262 PMC6484311

[12] Fu D, Lv X, Hua G, He C, Dong J, Lele SM, et al. YAP regulates cell proliferation, migration, and steroidogenesis in adult granulosa cell tumors. Endocr Relat Cancer. 2014;21(2):297–310. 24389730 10.1530/ERC-13-0339 PMC4222524

[13] Mizutani T, Orisaka M, Kawabe S, Morichika R, Uesaka M, Yoshida Y. YAP/TAZ-TEAD is a novel transcriptional regulator of genes encoding steroidogenic enzymes in rat granulosa cells and KGN cells. Mol Cell Endocrinol. 2023;559:111808. 36309205 10.1016/j.mce.2022.111808

[14] Shin JH, Lee G, Jeong MG, Kim HK, Won HY, Choi Y, et al. Transcriptional coactivator with PDZ-binding motif suppresses the expression of steroidogenic enzymes by nuclear receptor 4 A1 in Leydig cells. FASEB J. 2020;34(4):5332–47. 32067268 10.1096/fj.201900695RRRR

[15] Plewes MR, Talbott HA, Schott MB, Wood JR, Cupp AS, Davis JS. Unraveling the role of lipid droplets and perilipin 2 in bovine luteal cells. FASEB J. 2024;38:11 e23710. 10.1096/fj.202400260RR PMC11347014 38822676

[16] Summers AF, Pohlmeier WE, Sargent KM, Cole BD, Vinton RJ, Kurz SG, et al. Altered theca and cumulus oocyte complex gene expression, follicular arrest and reduced fertility in cows with dominant follicle follicular fluid androgen excess. PLoS ONE. 2014;9(10):e110683. 25330369 10.1371/journal.pone.0110683 PMC4199720

[17] Plewes MR, Przygrodzka E, Monaco CF, Snider AP, Keane JA, Burns PD, et al. Prostaglandin F2α regulates mitochondrial dynamics and mitophagy in the bovine Corpus Luteum. Life Sci Alliance. 2023. 10.26508/lsa.202301968. 10.26508/lsa.202301968 PMC10185813 37188480

[18] F. K. Trim Galore. Available from: https://www.bioinformatics.babraham.ac.uk/projects/trim_galore/.

[19] Dobin A, Davis CA, Schlesinger F, Drenkow J, Zaleski C, Jha S, et al. STAR: ultrafast universal RNA-seq aligner. Bioinformatics. 2013;29(1):15–21. 23104886 10.1093/bioinformatics/bts635 PMC3530905

[20] Love MI, Huber W, Anders S. Moderated estimation of fold change and dispersion for RNA-seq data with DESeq2. Genome Biol. 2014;15(12):550. 25516281 10.1186/s13059-014-0550-8 PMC4302049

[21] Tajima K, Yoshii K, Fukuda S, Orisaka M, Miyamoto K, Amsterdam A, et al. Luteinizing hormone-induced extracellular-signal regulated kinase activation differently modulates progesterone and androstenedione production in bovine theca cells. Endocrinology. 2005;146(7):2903–10. 15817663 10.1210/en.2005-0093

[22] Zhao B, Wei X, Li W, Udan RS, Yang Q, Kim J, et al. Inactivation of YAP oncoprotein by the Hippo pathway is involved in cell contact inhibition and tissue growth control. Genes Dev. 2007;21(21):2747–61. 17974916 10.1101/gad.1602907 PMC2045129

[23] Diaz FJ, Anderson LE, Wu YL, Rabot A, Tsai SJ, Wiltbank MC. Regulation of progesterone and prostaglandin F2α production in the CL. Mol Cell Endocrinol. 2002;191(1):65–80. 12044920 10.1016/s0303-7207(02)00056-4

[24] Christenson LK, Strauss JF. Steroidogenic acute regulatory protein (StAR) and the intramitochondrial translocation of cholesterol. Biochim Biophys Acta Mol Cell Biol Lipids. 2000;1529(1):175–87. 10.1016/s1388-1981(00)00147-5 11111087

[25] Lopez-Hernandez A, Sberna S, Campaner S. Emerging principles in the transcriptional control by YAP and TAZ. Cancers (Basel). 2021;13(16):4242. 34439395 10.3390/cancers13164242 PMC8391352

[26] Plewes MR, Hou X, Zhang P, Liang A, Hua G, Wood JR, et al. Yes-associated protein 1 is required for proliferation and function of bovine granulosa cells in vitro†. Biol Reprod. 2019;101(5):1001–17. 31350850 10.1093/biolre/ioz139 PMC6877782

[27] Lv X, He C, Huang C, Hua G, Chen X, Timm BK, et al. Reprogramming of ovarian granulosa cells by YAP1 leads to development of high-grade cancer with mesenchymal lineage and serous features. Sci Bull (Beijing). 2020;65(15):1281–96. 34888112 10.1016/j.scib.2020.03.040 PMC8654108

[28] Quirk SM, Cowan RG, Harman RM. Role of the cell cycle in regression of the corpus luteum. Reproduction. 2013;145(2):161–75. 23241346 10.1530/REP-12-0324

[29] Ardestani A, Lupse B, Maedler K. Hippo signaling: key emerging pathway in cellular and whole-body metabolism. Trends Endocrinol Metab. 2018;29(7):492–509. 29739703 10.1016/j.tem.2018.04.006

[30] Honda D, Okumura M, Chihara T. Crosstalk between the mTOR and hippo pathways. Dev Growth Differ. 2023;65(6):337–47. 37209252 10.1111/dgd.12867

[31] Abdou HS, Bergeron F, Tremblay JJ. A cell-autonomous molecular cascade initiated by AMP-activated protein kinase represses steroidogenesis. Mol Cell Biol. 2014;34(23):4257–71. 25225331 10.1128/MCB.00734-14 PMC4248749

