# Supplementary material for: Luteinizing hormone activates the Hippo pathway to promote progesterone synthesis in bovine luteal cells
Source: Cell Commun Signal. 2026 May 2;24:367. doi: 10.1186/s12964-026-02917-w (PMC13281590; doi:10.1186/s12964-026-02917-w)

Supporting Information Figure 1. Ratio of p-YAP1Ser127 and p-YAP1Ser397 to total YAP1 in differentiated TC and GC.

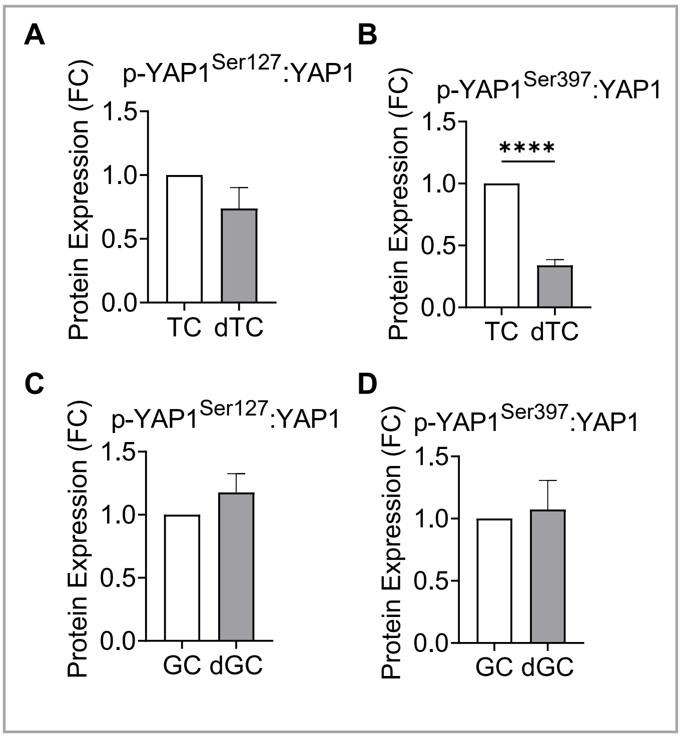

Supplement: Supplementary file 1 — Supplementary Material 1: Supporting Figure 1. Ratio of p-YAP1Ser127 and p-YAP1Ser397 to total YAP1 in differentiated TC and GC. Bovine theca and granulosa cells were cultured under luteinizing conditions for 96 h.Quantified ratio of p-YAP1Ser127 and p-YAP1Ser397 to total YAP1 protein expression shown as fold changein differentiated TC and differentiated GC, respectively. ACTB, loading control. Data means are ± SEM. Paired t-test: ****P < 0.0001. [file 12964_2026_2917_MOESM1_ESM.pdf]
