# Supplementary figures and images for: Luteinizing hormone activates the Hippo pathway to promote progesterone synthesis in bovine luteal cells
Source: Cell Commun Signal. 2026 May 2;24:367. doi: 10.1186/s12964-026-02917-w (PMC13281590; doi:10.1186/s12964-026-02917-w)

Supporting Information Figure 2. The shift in phosphorylation of YAP1 upon LH/FSK treatment.

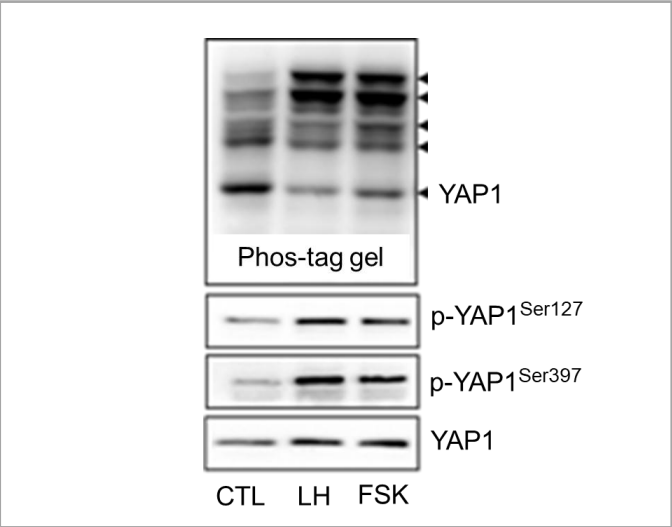

Supplement: Supplementary file 2 — Supplementary Material 2: Supporting Figure 2. The shift in phosphorylation of YAP1 upon LH/FSK treatment. Bovine small luteal cells were treated with LHor forskolinfor 30 minutes. Representative Phos-tag SDS-PAGE showing a mobility shift of YAP1 in response to LH and FSK, indicative of increased phosphorylation. [file 12964_2026_2917_MOESM2_ESM.pdf]

Supporting Information Figure 3. Subcellular localization of Hippo effectors in fold change.

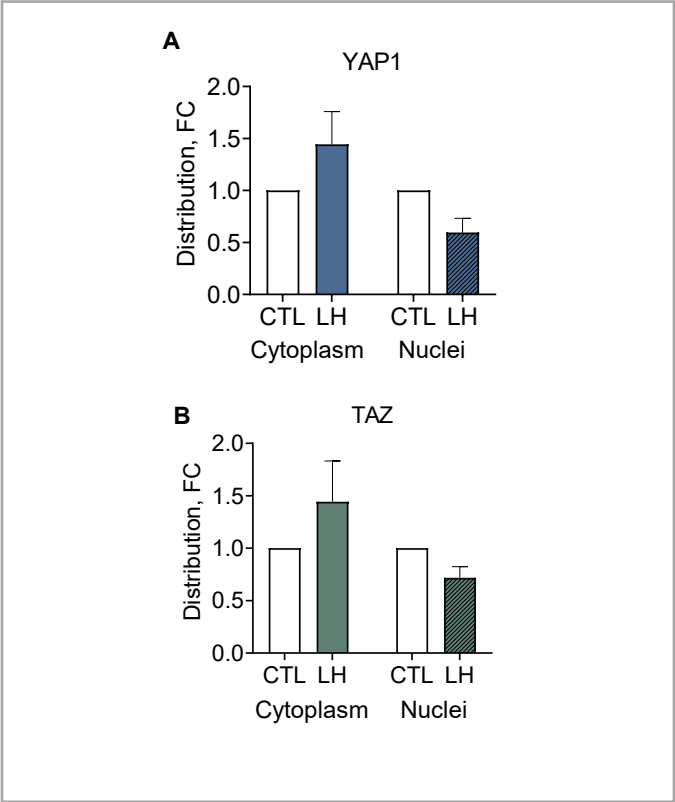

Supplement: Supplementary file 3 — Supplementary Material 3: Supporting Figure 3. Subcellular localization of Hippo effectors. Bovine small luteal cells were treated with or without LHfor 1 h, followed by subcellular fractionation.The graphs present total YAP1 and TAZ FC distribution in cytoplasmic and nuclear fractions, following LH treatment. [file 12964_2026_2917_MOESM3_ESM.pdf]

Supporting Information Figure 7. Successful siRNA-mediated YAP1/TAZ knockdown.

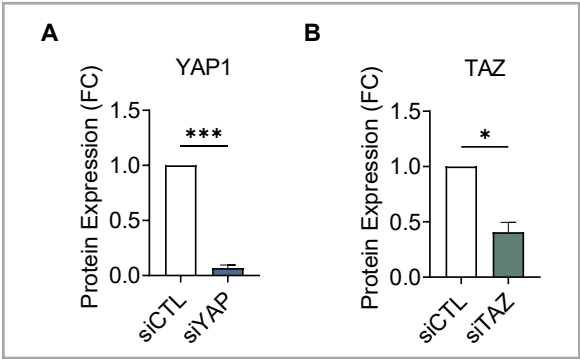

Supplement: Supplementary file 7 — Supplementary Material 7: Supporting Figure 7. Successful Si-mediated YAP1/TAZ knockdown. Bovine small luteal cells were transfected with siRNAs targeting YAP1 or TAZ.YAP1 and TAZ Knockdown efficiencies were 93.1% ± 2.7% and 59.4% ± 8.9%, respectively. ACTB, loading control. Data means are ± SEM. Paired t-test: *P < 0.05, ***P < 0.001. [file 12964_2026_2917_MOESM7_ESM.pdf]
