# Supplementary material for: Luteinizing hormone activates the Hippo pathway to promote progesterone synthesis in bovine luteal cells
Source: Cell Commun Signal. 2026 May 2;24:367. doi: 10.1186/s12964-026-02917-w (PMC13281590; doi:10.1186/s12964-026-02917-w)

Supporting Information Figure 4. Downregulation of YAP1/TAZ target genes following LH-induced inhibition.

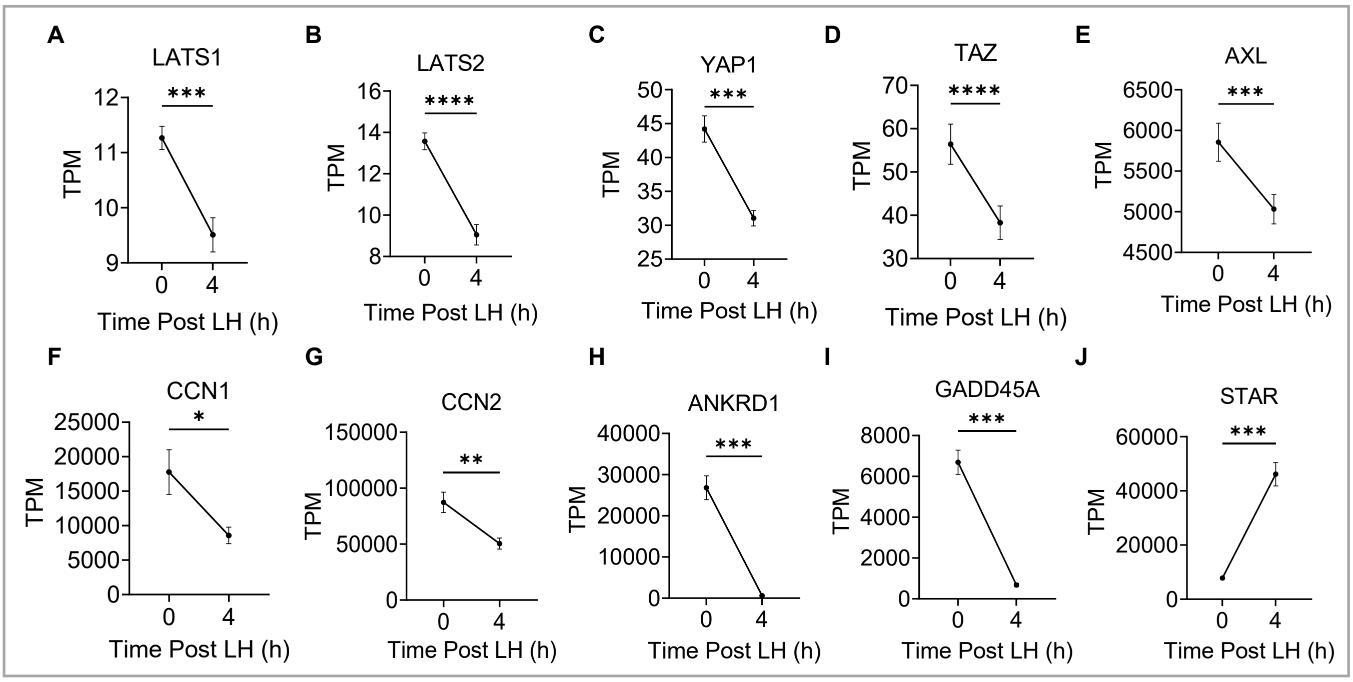

Supplement: Supplementary file 4 — Supplementary Material 4: Supporting Figure 4. Downregulation of YAP1/TAZ target genes following LH-induced inhibition. Bovine small luteal cells were treated with LHfor 0 or 4 h, followed by RNA-seq.Representative graphs show downregulation of Hippo pathway components andwell-known YAP1 and TAZ target genes.Upregulation of STAR. Data means are ± SEM. Paired t-test: *P < 0.05, **P < 0.01, ***P < 0.001. [file 12964_2026_2917_MOESM4_ESM.pdf]
