# Supplementary material for: Luteinizing hormone activates the Hippo pathway to promote progesterone synthesis in bovine luteal cells
Source: Cell Commun Signal. 2026 May 2;24:367. doi: 10.1186/s12964-026-02917-w (PMC13281590; doi:10.1186/s12964-026-02917-w)

Supporting Information Figure 5. LATS1/2 inhibition by TRULI showed no significant change in progesterone output.

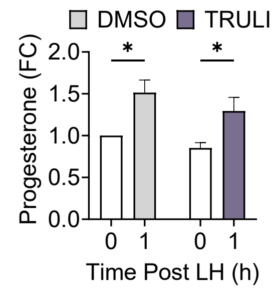

Supplement: Supplementary file 5 — Supplementary Material 5: Supporting Figure 5. LATS1/2 inhibition by TRULI showed no significant change in progesterone output. Bovine small luteal cells were pre-treated for 1 h with TRULI, then stimulated with or without LHfor 1 h. Progesterone levels measured by ELISA. Data represent mean ± SEM. Two-way ANOVA with uncorrected Fisher’s LSD: *P < 0.05. [file 12964_2026_2917_MOESM5_ESM.pdf]
