# Supplementary material for: Luteinizing hormone activates the Hippo pathway to promote progesterone synthesis in bovine luteal cells
Source: Cell Commun Signal. 2026 May 2;24:367. doi: 10.1186/s12964-026-02917-w (PMC13281590; doi:10.1186/s12964-026-02917-w)

Supporting Information Figure 6. Basal progesterone production in large luteal cells overexpressing YAP1S127A or TAZS89A compared to GFP control.

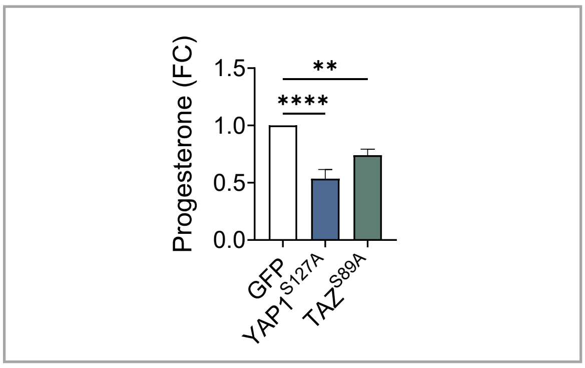

Supporting Information Figure 7. Successful siRNA-mediated YAP1/TAZ knockdown.

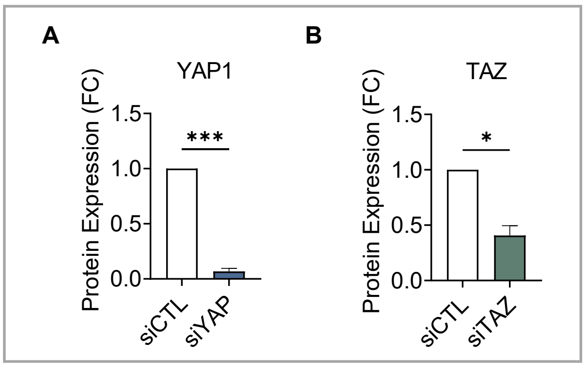

Supplement: Supplementary file 6 — Supplementary Material 6: Supporting Figure 6. Constitutively active mutant YAP1 and TAZ suppress basal progesterone production in large luteal cells. Bovine large luteal cells were infected with adenovirusesoverexpressing GFP, constitutively active YAP1S127A or constitutively active TAZS89A for 48 h. Progesterone levels measured by ELISA. Data represent mean ± SEM. One-way ANOVA with Dunnett’s multiple comparisons: **P < 0.01, ****P < 0.0001. [file 12964_2026_2917_MOESM6_ESM.pdf]
